# Impact of Selected Bacterial and Viral Toll-like Receptor Agonists on the Phenotype and Function of Camel Blood Neutrophils

**DOI:** 10.3390/vetsci10020154

**Published:** 2023-02-14

**Authors:** Jamal Hussen, Mayyadah Abdullah Alkuwayti, Baraa Falemban, Sameer M. Alhojaily, Salma Al Adwani, El Awad El Hassan, Abdullah IA Al-Mubarak

**Affiliations:** 1 Department of Microbiology, College of Veterinary Medicine, King Faisal University, Al-Ahsa 31982, Saudi Arabia; 2 Department of Biological Sciences, College of Science, King Faisal University, Al Ahsa 31982, Saudi Arabia; 3 Department of Biomedical Sciences, College of Veterinary Medicine, King Faisal University, Al-Ahsa 31982, Saudi Arabia; 4 Agricultural and Veterinary Training and Research Station, King Faisal University, Al-Ahsa 31982, Saudi Arabia; 5 Department of Animal & Veterinary Sciences, Sultan Qaboos University, Muscat 123, Oman

**Keywords:** TLR agonists, camel, neutrophils, immune response, ROS, phagocytosis, apoptosis

## Abstract

**Simple Summary:**

Polymorphonuclear neutrophils belong to the first line of defense with major contributions to the early innate recognition and elimination of pathogens. The development of effective vaccines for camel infectious diseases will profit from understanding the interaction mechanisms between innate immune cells and pathogen-associated molecular patterns (PAMPs), such as toll-like receptor (TLR) ligands. The present study investigated the impact of the TLR ligands LPS, Pam3CSK4, R848 (Resiquimod), and Poly IC on the phenotype and function of camel blood neutrophils. The analysis of stimulation-induced changes in neutrophil cell size, their phagocytosis and ROS production activity, the expression of cell surface antigens, and cell viability identified agonist-specific modulatory effects on camel blood neutrophils.

**Abstract:**

Innate recognition of pathogens depends on the interaction between microbial structures known as pathogen-associated molecular patterns (PAMPs) and pattern recognition receptors (PRRs) in host cells. Toll-like receptors (TLR) are among the most important PRRs being expressed on and in a wide range of immune cell types. Studies on the interaction mechanisms between different pathogen species and the immune system of the dromedary camel are still scarce. The present study aimed to investigate the immunomodulatory effect of synthetic bacterial and viral TLR ligands on some phenotypic properties and selected functions of neutrophils purified from dromedary camel blood. Neutrophils were separated from camel blood (n = five animals) and were stimulated in vitro with the TLR ligands LPS, Pam3CSK4, R848 (Resiquimod), and Poly IC or were left without stimulation. Stimulation with the protein kinase C activator phorbol 12-myristate 13-acetate (PMA) was used as a positive control stimulation. Shape change, phagocytosis activity, ROS production, the expression of cell surface markers, and cell vitality were compared between stimulated and non-stimulated cells. With exception of the TLR3 agonist Poly IC, all TLR ligands used showed the potential to stimulate camel neutrophils resulting in increased cell size and the upregulation of CD18 and CD14 on their surface. Similarly, the phagocytosis activity of camel neutrophils was significantly improved after priming with all TLR ligands, except Poly IC, which, in contrast, resulted in a reduced percentage of phagocytosis-positive cells. In contrast to stimulation with PMA, which induced a significant ROS production in camel neutrophils, none of the TLR ligands used stimulated ROS generation in neutrophils. Only stimulation with Pam3CSK4 increased the expression of MHCII molecules on camel neutrophils, resulting in an expanded MHCII^high^ fraction within camel neutrophils. Our study indicates selective immunomodulating effects of TLR agonists on purified camel neutrophils without affecting their vitality.

## 1. Introduction

Neutrophils are key players in the early immune response to pathogens. They contribute to pathogen recognition, elimination, and subsequent activation of the innate and adaptive immune response [1,2]. Pathogen detection and sensing is the first step before triggering the innate immune response and the subsequent initiation of the adaptive immune response [3,4,5,6,7,8]. Innate sensing is mediated by several pattern recognition receptors (PRRs) that recognize distinct structures associated with infection or tissue injury, known as pathogen-associated molecular patterns (PAMPs) and danger-associated molecular patterns (DAMPs), respectively [3,9]. Toll-like receptors (TLR) are among the most important members of PRRs. They are expressed both on the cell surface and in the intracellular compartment [10,11,12] and can recognize bacterial and viral PAMPs and induced DAMPs [4,5,6,7,8,13].

The endotoxin lipopolysaccharide (LPS) is a component of gram-negative bacteria [14,15] that is recognized by TLR4, CD14, LBP, and the adaptor protein MD-2 [16,17,18]. The TLR1/2 ligand Pam3CSK4 is a PAMP of gram-positive bacteria [19,20]. The imidazoquinoline resiquimod (R848) is a dual TLR8 and TLR7 synthetic agonist used to mimic innate responses to RNA viruses [20].

Key effector functions of neutrophils include the uptake and killing of microbes by production of reactive oxygen species (ROS), formation of neutrophil extracellular traps (NETs), and degranulation [1,2]. Studies in several species revealed immunomodulatory effects of TLR activation on several effector functions of neutrophils, such as phagocytosis and ROS production, degranulation, and NETs formation [21].

Studies on the immune response of camels to bacterial and viral pathogens are still scarce, and the interaction between bacterial and viral TLR ligands and camel neutrophils has not been investigated so far. The aim of the present work was to investigate the immunomodulatory effects of selected TLR agonists representing responses to bacterial and viral PAMPs on the phenotype and function of camel neutrophils.

## 2. Animals, Materials, and Methods

### 2.1. Animals and Sampling

Five clinically healthy male dromedary camels were involved in the study and used for collection of blood samples. The camel farm was located in the Al-Ahsa region, Saudi Arabia. Blood samples were collected from the jugular vein into vacutainer tubes containing the anticoagulation agent EDTA. The samples were kept on ice and delivered to the laboratory within 1 hour of blood collection. Separation of neutrophils from blood specimens was undertaken within 2 hours of sample collection. The study was approved by the Ethics Committee of King Faisal University (approval no KFU-REC-2021-DEC-EA000326).

### 2.2. Isolation of Neutrophils from Camel Blood

Camel blood neutrophils were separated as previously described for bovine neutrophils [22]. Blood (15 mL) was diluted with PBS (1:2) and the mixture was layered on 15 mL Ficoll–Isopaque 1.077 g/mL (Lymphoprep™. Stemcell Technologies, Burnaby, Canada) in a 50 mL sterile conical tube. The tubes were centrifuged at room temperature (RT) 20 °C for 30 min at 800× *g* without a break. After centrifugation, the interphase containing mononuclear cells was removed and the PMN-erythrocyte-rich phase was used for PMN separation after erythrolysis. The lysis of red blood cells was achieved through a short incubation in hypotonic solution and restoring of tonicity. For this, distilled water (20 mL) was added to the cells for 20 sec and a similar volume of double concentrated PBS was then added to the cells to restore the osmotic pressure. The lysis cycle was repeated until all erythrocytes were completely lysed (usually after two to three cycles) and the pure white pellet of PMNs was suspended in Hank’s balanced salt solution (HBSS; MOLEQULE-ON, Auckland, New Zealand) at 1 × 10^7^ cells/mL. Neutrophil viability was measured after incubation with 2 µg/mL of propidium iodide (Calbiochem, Germany), and it was always above 97%. Neutrophil purity (minimal contamination with mononuclear cells) was always above 95%.

### 2.3. Reagents

Lipopolysaccharide (*E. coli* serotype 0111:B4; tlrl-eblps), Pam3CSK4 (tlrl-pms), R848 (Resiquimod; tlrl-r848), and Poly IC (tlrl-pic) were purchased from Invivogen (San Diego, USA). PMA was purchased from Calbiochem (Merck Millipore, Darmstadt, Germany).

### 2.4. In Vitro Stimulation of Purified Neutrophils with Synthetic Toll-like Receptor Ligands

In vitro stimulation of purified blood neutrophils was performed based on a previously published method for bovine neutrophils with minor changes [20,23]. Isolated neutrophils were suspended in HBSS at a final concentration of 1 × 10^7^ cells/mL. For the in vitro priming of neutrophils, 1 × 10^6^ cells in 100 µL medium were incubated for 30 min at 37 °C and 5% CO2 with 1 μg/mL of the TLR4-ligand LPS, 1 μg/mL of the TLR2/1-ligand Pam3CSK4, 0.2 µg/mL of the TLR7/8-ligand R848 (Resiquimod), 10 µg/mL of the TLR3-ligand Poly IC, or 10 ng/mL of the protein kinase C activator phorbol 12-myristate 13-acetate (PMA), or only HBSS medium without stimulants. The concentrations of the stimulants were chosen based on previously described concentrations for the stimulation of bovine cells [20].

### 2.5. Stimulation-Induced Neutrophil Cell Size Change

The change in cell size was determined by flow cytometric measurement of alterations in the forward light scatter (FSC) value of the neutrophils (Accuri C6 flow cytometer; BD Biosciences), as described previously [23]. Neutrophils in 100 µL HBSS medium (1 × 10^6^ cells) were incubated for 30 min at 37 °C and 5% CO2 with 1 μg/mL of LPS, 1 μg/mL of Pam3CSK4, 0.2 µg/mL of R848, 10 µg/mL of Poly IC, or 10 ng/mL of PMA. Mean FSC values for stimulated neutrophils were compared with mean FSC values of non-stimulated cells in the medium control (cells in HBSS medium without stimulation). Overlapping histograms were generated using the C flow software (BD Biosciences) to make graphical comparisons.

### 2.6. Impact of TLR Priming on Bacterial Phagocytosis by Camel Neutrophils

The impact of TLR priming on the phagocytosis activity of purified neutrophils was analyzed using flow cytometry. [24]. For this, heat-killed *S. aureus* (Pansorbin) was purchased from Calbiochem (Nottingham, UK) and labeled with fluoresceinisothiocyanate (FITC) according to the manufacturer’s protocol (fluoresceinisothiocyanate labeling kit, Sigma-Aldrich, St. Louis, MO, USA). Briefly, heat-killed *S. aureus* were washed twice with PBS (14,000× *g* and 4 °C for 5 min), resuspended in PBS, and incubated for 30 min with FITC (0.5 mg/mL) in 1 mL of PBS at RT in the dark. Subsequently, bacteria were washed 3 times in PBS (14.000× *g* for 5 min) to remove the unbound FITC molecules. Pelleted bacteria were resuspended in PBS and adjusted to 2 × 10^8^ bacteria/mL and stored in aliquots at –80 °C. Control and stimulated neutrophils in 100 µL HBSS medium containing 1 × 10^6^ cells were incubated with *S. aureus*-FITC in a ratio of 30 bacteria for each neutrophil for 30 min at 37 °C and 5% CO2. Finally, the unbound bacteria were removed by washing the plate in HBSS medium (centrifugation at 300× *g* for 3 min), and the cells were analyzed by flow cytometry after resuspension in 150 μL of HBSS medium.

### 2.7. Generation of Reactive Oxygen Species by Neutrophils

The production of reactive oxygen species (ROS) by camel neutrophils was analyzed using the ROS dye dehydrorohdamin-123 (DHR-123) and flow cytometry. Control and TLR-stimulated neutrophils (1 × 10^6^ in 100 µL HBSS medium) were stained with DHR-123 for 15 min at 37 °C and 5% CO2. The DHR dye was added to the cells 15 min after the addition of stimulants. After stimulation, the cells were washed (3 min at 300× *g*) in HBSS and were then suspended in 150 µL HBSS. Stained cells were measured on the Accuri C6 flow cytometer. The DHR-123 dye changes to the green fluorescing rhodamine in the presence of ROS metabolites. ROS response was measured as the increase in the green fluorescence (mean FL-1) of neutrophils.

### 2.8. Cell Surface Antigen Expression on Neutrophils

Control and TLR-stimulated neutrophils (1 × 10^6^ cells in 100 µL HBSS per well) were centrifuged (300× *g* for 3 min) and incubated in flow cytometry buffer (PBS containing bovine serum albumin (5 g/L; Sigma) andNaN3 (100 mg/L; Sigma)) with the following combinations of mouse monoclonal antibodies [25,26,27,28,29]: anti-bovine CD14 (IgG1; clone CAM36A) with anti-MHCII (IgG2a; clone TH81A5); anti-CD172a (IgG1; clone DH59b) with anti-CD44 (IgG2a; clone LT41A); or FITC-conjugated mouse anti CD18 and PE-conjugated anti CD11a. After incubation of cells and primary antibodies for 15 min at 4 °C, the cell suspension was washed by the addition of 150 µL cold staining buffer per well of the plate and centrifugation of the plate for 3 min at 300× *g* and 4 °C. For the identification of primary antibodies bound to the cells, a second staining step followed with the addition of secondary goat antibodies against the mouse Ig isotypes IgG1 and IgG2a was performed. The secondary antibodies that were conjugated with different fluorochromes were added to the wells for a further 15 min at 4 °C in the dark. A final washing step was performed to remove unbound secondary antibodies (washing in 150 µL of cold staining buffer and centrifugation at 300× *g* and 4 °C for 3 min). Finally, the cells were taken in 150 µL staining buffer and kept on ice until (usually within 1 hour) analysis on the Accuri C6 flow cytometer (BD Biosciences). Staining with mouse Ig isotype controls was also performed.

### 2.9. Neutrophils Vitality Assay

Cell apoptosis and necrosis were analyzed using the annexin V-FITC apoptosis staining/detection kit following the manufacturer’s protocol (Abcam; ab14085). The kit includes both annexin V-FITC and PI staining to detect cell apoptosis and necrosis, respectively [23]. The plates containing non-stimulated and stimulated camel neutrophils (1× 10^6^ cells in 100µL HBSS cell culture medium) were centrifuged (300× *g* for 3 min at RT) and 100µL of KIT buffer containing 1:100 annexin V-FITC and 1:100 PI were added to the cells. After incubation for 5 min at RT in the dark, the cells were analyzed on the flow cytometer (BD Accuri C6 flow cytometer). Apoptotic cells (annexin V positive/ PI negative) were differentiated from necrotic (annexin V positive/PI positive) and viable cells (annexin V negative/PI negative) based on their FL1 and FL2 fluorescence.

### 2.10. Statistical Analysis

The statistical software GraphPad Prism (v5, San Diego, CA, USA) was used for data normality testing and the comparison between the in vitro stimulation set-ups. The comparison between non-stimulated neutrophils and neutrophils stimulated with LPS, Pam3C4K, R848, Poly IC, or PMA was analyzed by the ANOVA test (1-factorial analysis of variance) in combination with Bonferroni’s multiple comparison test (*p*-value less than 0.05 indicates significant differences). The analysis results were presented as mean ± standard error of the mean (SEM) and graphs were produced using the same program.

## 3. Results

### 3.1. Impact of Toll-like Receptor Ligands on Cell Size of Stimulated Neutrophils

**Figure 1:**
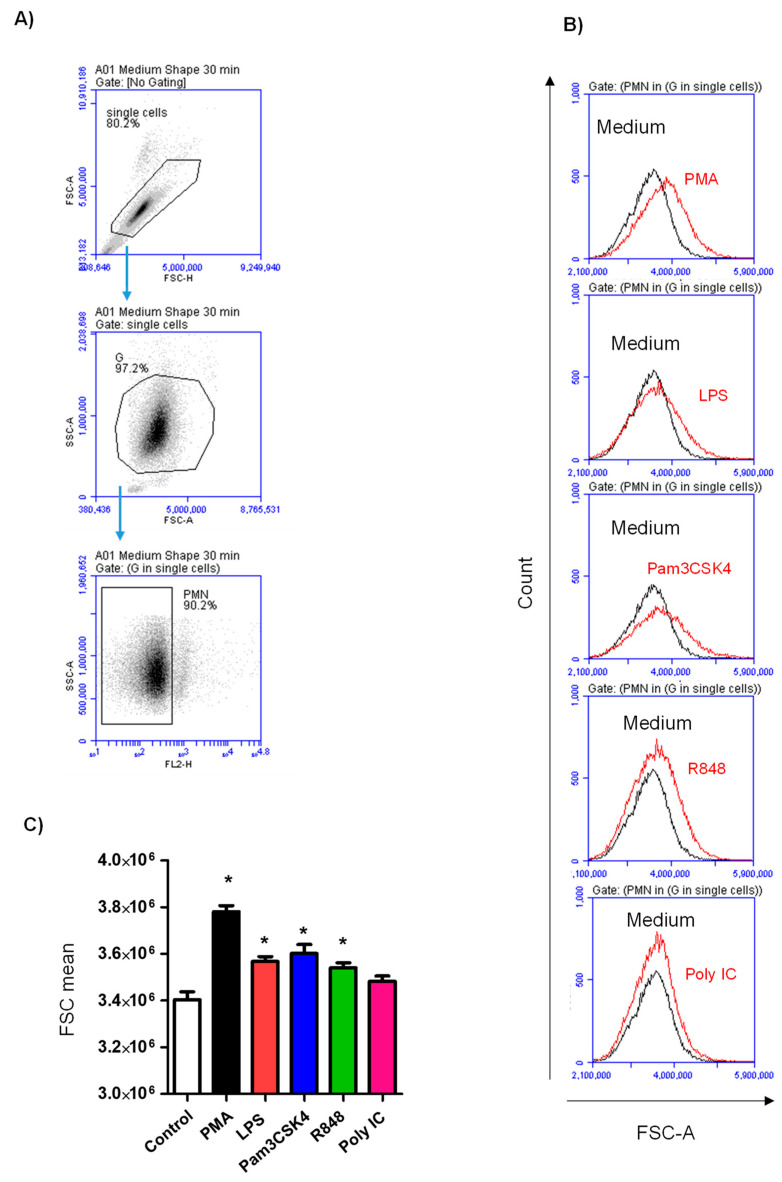
TLR stimulation-induced change in neutrophil cell size. (**A**) Single cells were identified in an FSC-H/FSC-A gate. Neutrophils (PMN) were identified within the granulocyte population (G) based on their lower auto-fluorescence in the FL-2 channel compared to eosinophilic granulocytes. (**B**) Overlapping histograms showing the change in FSC-A of stimulated neutrophils (red line) compared with control neutrophils (black lines). (**C**) Mean FSC-A values were calculated for control and stimulated neutrophils and presented graphically as mean and SEM. * indicates significant change in the FSC-A between the means of stimulated and non-stimulated cells with *p* values less than 0.05.

Stimulation-induced shape change in neutrophils was analyzed by flow cytometric analysis of forward scatter signals. Overlapping histograms of gated neutrophils show the change in cell size after TLR ligand stimulation (Figure 1A,B). Stimulation with PMA as a positive control stimulator or any of the TLR ligands, with the exception of Poly IC, induced a significant increase in the cell size of neutrophils (*p* < 0.05) in comparison to cells in the medium control. The strongest shape change was observed after stimulation with PMA (Figure 1A).

### 3.2. TLR Ligands Differently Modulate the Phagocytosis Activity of Camel Neutrophils

**Figure 2:**
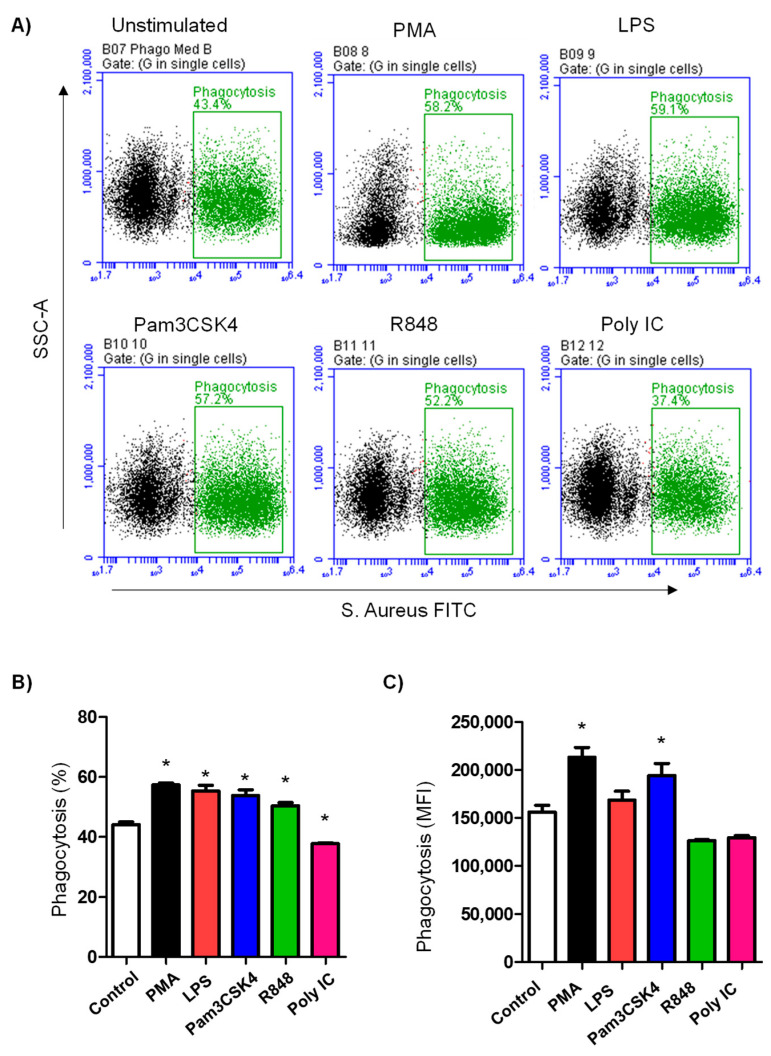
Analysis of neutrophils phagocytosis activity by flow cytometry. (**A**) Non-stimulated and stimulated camel neutrophils were incubated with FITC-*S. aureus* and analyzed on the flow cytometer. Phagocytic neutrophils were identified as cells with higher fluorescence in the FL-1. The fraction of phagocytic cells (**B**) as well as their staining intensity (MFI) (**C**) were presented for stimulated and non-stimulated neutrophils. * indicates a *p* value > 0.05.

Phagocytosis by camel neutrophils was determined using flow cytometry after incubating the cells with *S. aureus* bacteria conjugated with FITC (Figure 2A). For non-stimulated cells in the medium control, the percentage of phagocytosis-positive neutrophils was 44.1 ± 0.8% of total cells. Pre-stimulation of camel neutrophils with PMA (56.2 ± 1.0%), LPS (53.1 ± 1.8%), Pam3CS4K (50.8 ± 1.7% of total cells), or R848 (48.4 ± 1.0%) significantly increased (*p* < 0.05) the percentage of phagocytosis-positive cells, while pre-stimulation with Poly IC (37.2 ± 0.3% of total cells) reduced (*p* < 0.05) the percentage of phagocytic neutrophils (Figure 2B). The mean fluorescence intensity (MFI) of phagocytic cells that reflects the phagocytosis capacity of each neutrophil cell (indicating the number of bacterial particles ingested by each neutrophil) was, however, only increased for PMA- or Pam3CS4K-stimulated cells (Figure 2C).

### 3.3. ROS Production in Neutrophils after TLR Stimulation

**Figure 3:**
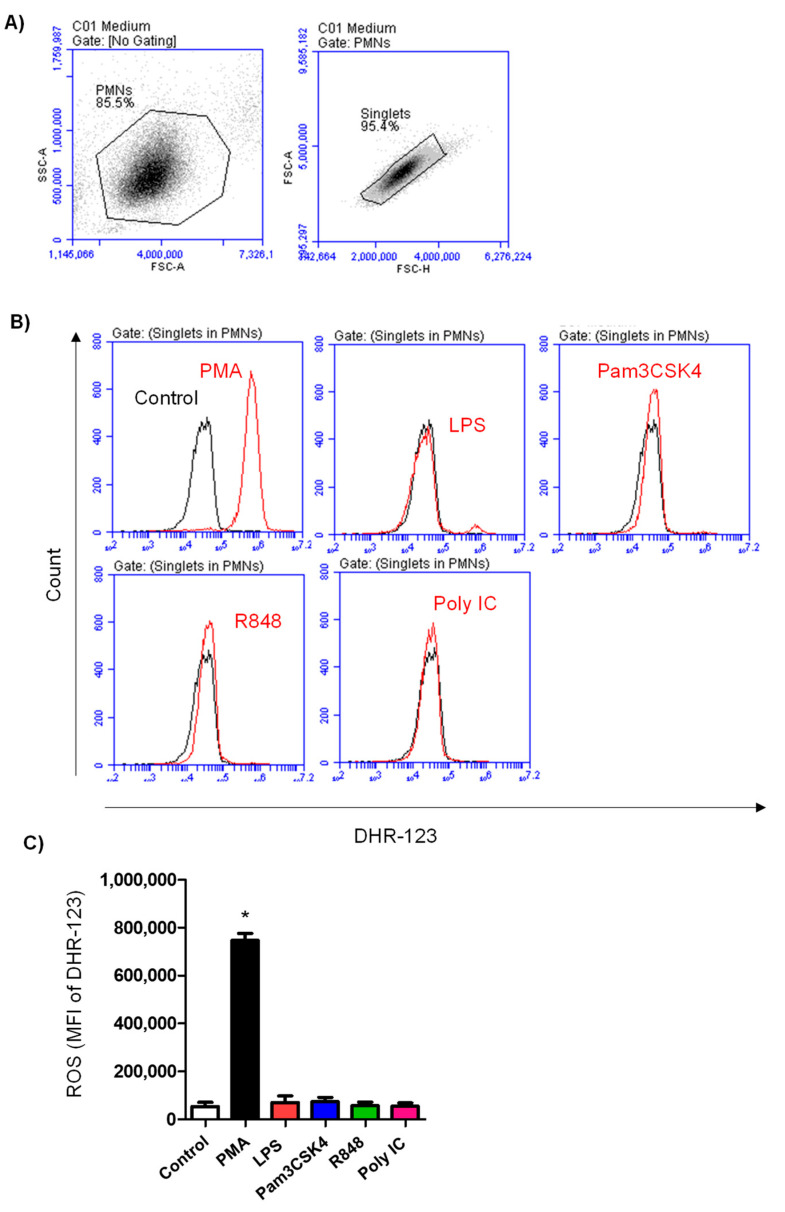
Analysis of ROS generation in neutrophils. (**A**) Non-stimulated and stimulated camel neutrophils were incubated with DHR-123 and analyzed on the flow cytometer. After gating on single neutrophils (**A**), overlapping histograms (**B**) were generated to show the change in DHR fluorescence of stimulated neutrophils (red line) compared with control neutrophils (black lines). (**C**) Mean FL-1 values were measured for control and stimulated neutrophils and presented as mean ± SEM. * indicates significant change in the FSC-A between the means of stimulated and non-stimulated cells with *p* values less than 0.05.

Production of ROSs was estimated by the measurement of green fluorescence intensity of stimulated neutrophils labeled with the fluorescent dye DHR 123 (Figure 3A,B). Only stimulation with PMA (MFI of DHR 746,927 ± 28,936 versus 53,785 ± 17,473 for non-stimulated cells) resulted in a 13-fold increase in the mean fluorescence intensity of DHR-labeled neutrophils, indicating a significant (*p* < 0.05) stimulation-induced ROS formation by neutrophils (Figure 3C). In contrast to this, none of the TLR ligands used stimulated (*p* > 0.05) ROS formation in camel neutrophils (Figure 3C).

### 3.4. TLR Ligands Modulate the Expression of Some Neutrophil Cell-Surface Antigens

**Figure 4:**
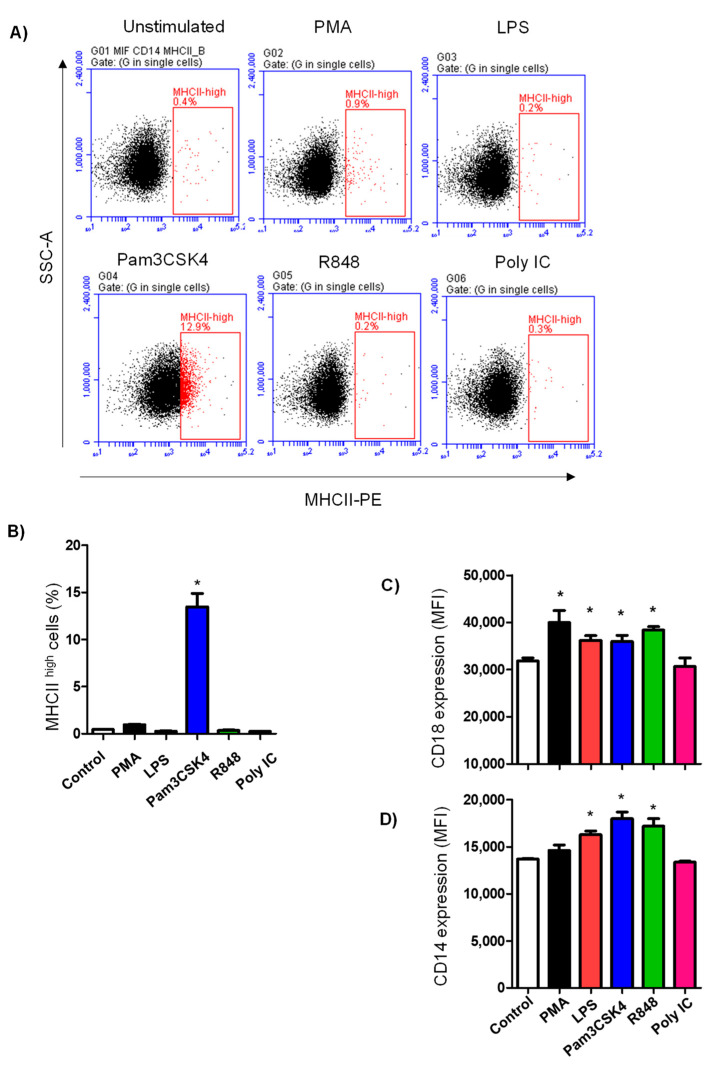
Analysis of the abundance of selected neutrophil cell-surface antigens. Neutrophils (after stimulation) were stained with anti-CD14, anti-MHCII, anti-CD172a, anti-CD44, anti-CD11a, and anti-CD18 antibodies, and the stained cells were analyzed by flow cytometry. (**A**) Representative density plots showing the expression of MHCII molecules on control and stimulated neutrophils. (**B**) The percentage of MHCII^high^ neutrophils within the neutrophil population. Mean fluorescence intensity (MFI) of neutrophils stained with antibodies to CD18 (**C**) and CD14 (**D**) were calculated and presented graphically as mean ± SEM. * indicates significant difference in the FSC-A means between stimulated and non-stimulated cells with *p* values less than 0.05.

The stimulation-induced change in the abundance of the molecules CD14, MHCII, CD44, CD172a, CD11a, and CD18 was analyzed in camel neutrophils by flow cytometry. Only stimulation with Pam3CS4K induced a significant increase (*p* < 0.05) in the percentage of MHCII^high^ neutrophils (13.4 ± 0.3%) in comparison to control cells (0.4 ± 0.03% of total cells) (Figure 4A,B). With the exception of Poly IC, all TLR ligands enhanced the abundance of CD14 (Figure 4C) and CD18 (Figure 4D) on camel neutrophils (*p* < 0.05). Stimulation with PMA enhanced CD18 expression but did not affect the expression of CD18. There was no change in the expression density of CD44, CD11a, or CD172a on camel neutrophils after stimulation with PMA or the different TLR ligands (data not shown).

### 3.5. Stimulation with TLR Ligands Does Not Affect Cell Vitality of Camel Neutrophils

**Figure 5:**
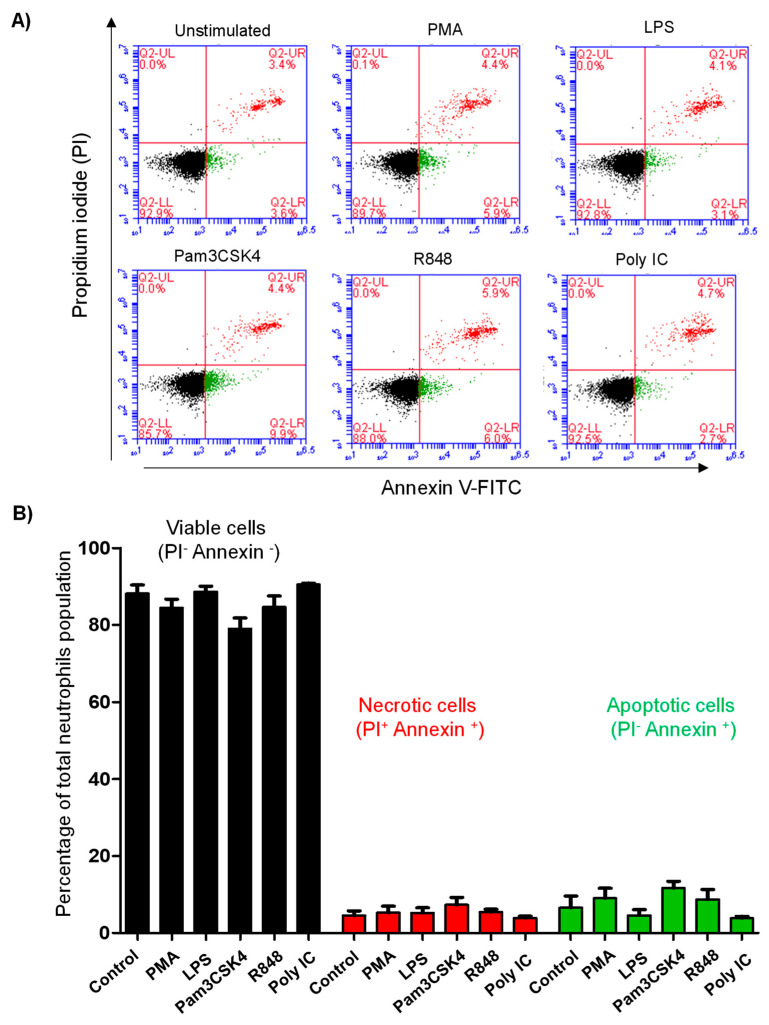
Flow cytometric analysis of cell vitality of camel neutrophils. Control and stimulated neutrophils were labeled with the cell apoptosis dye annexin V-FITC and the cell necrosis dye propidium iodide (PI) and analyzed by flow cytometry. (**A**) Representative dot plots of annexin V-FITC against PI fluorescence showing the identification of necrotic (PI^+^ /annexin V^+^), apoptotic (PI^−^/annexin V^+^), and live neutrophils (PI^-^/annexin V^−^) upon excitation at 488 nm. (**B**) The percentage of necrotic, apoptotic, and viable neutrophils was calculated and presented as mean ± SEM. * indicates significant change in the FSC-A between the means of stimulated and non-stimulated cells with *p* values less than 0.05.

Neutrophil cell vitality was evaluated by the analysis of cell necrosis and apoptosis using flow cytometry and combined staining with annexin V and propidium iodide (PI). Cell necrosis was detected based on positive staining with the DNA-binding dye PI and the phosphatidylserine-binding protein annexin V, while single staining with annexin V was used to identify apoptotic cells (Figure 5A,B). Neither stimulation with PMA nor with the TLR ligands resulted in a significant change (*p* > 0.05) in the fractions of viable, necrotic, or apoptotic neutrophils (Figure 5C).

## 4. Discussion

Polymorphonuclear neutrophils belong to the first effector immune cells acting against pathogens and contribute effectively to the early innate recognition and elimination of microbes [30,31,32,33,34]. Innate recognition of pathogens depends on the interaction between microbial structures known as pathogen-associated molecular patterns (PAMPs) and pattern recognition receptors (PRRs) in innate immune cells [35,36]. Toll-like receptors (TLR) are among the most important PRRs being expressed on and in many immune cells [37]. Studies on the interaction between TLR and their agonists in camel immune cells are still scarce. The present work analyzed the immunomodulatory effects of the TLR agonists LPS, Pam3CSK4, R848 (Resiquimod), and Poly IC on selected phenotypic and functional properties of camel neutrophils.

The expression of several TLRs has been reported previously in bovine and human neutrophils [23,38]. In the present study, all TLR ligands used, with exception of the TLR3 ligand Poly IC, showed the potential to stimulate camel neutrophils resulting in a significant shape change (increased forward scatter (FSC) values). In addition, the upregulation of CD14 and CD18 (two neutrophil activation markers [39]) on the surface of TLR ligand-stimulated neutrophils also supports this finding. Although not proven using TLR-specific antibodies (due to the unavailability of camel-specific antibodies), the reactivity of neutrophils toward LPS, Pam3CSK4, and R848 and the lack of their response to Poly IC indicate the expression of TLR4, TLR2/1, and TLR7/8, but the lack of TLR3 in camel neutrophils. Further studies are, however, required to confirm their expression using TLR-specific antibodies or using fluorochrome-labeled TLR-specific agonists.

Studies on human [40,41] and bovine [42] neutrophils revealed different functional changes after the ligation of TLRs by their specific agonists. Effector functions of neutrophils include the phagocytosis and killing of microbes by oxygen-dependent and independent mechanisms. To determine whether priming with TLR ligands may modulate the response of camel neutrophils to a subsequent bacterial stimulation, we analyzed the phagocytosis activity of TLR ligand-primed neutrophils. The results of the present study revealed an enhancing effect of priming with LPS, Pam3CSK4, R848, but not Poly IC on the phagocytosis activity of neutrophils without affecting their vitality (apoptosis or necrosis). These results are in line with previous studies on human neutrophils, where TLR stimulation resulted in the enhancement of their phagocytosis activity [40]. Further studies are needed to identify the mechanism behind the observed negative effect of priming with Poly IC on the phagocytosis activity of camel neutrophils.

The lack of an ROS-inducing effect of TLR priming on camel neutrophils is in contrast to the reported increase in ROS response of human neutrophils after stimulation with the same TLR agonists. In the latter study, ROS production was mainly produced after a subsequent fMLF stimulation of TLR ligand-primed neutrophils. The requirement of two activating stimuli for an effective activation of neutrophils has been demonstrated in a recent report [43]. The priming effect of TLR ligands on the response of camel neutrophils to ROS-inducing stimulants should be evaluated in further studies.

Recent studies reported the capacity of neutrophils to express major histocompatibility molecules class (MHC)-II on their surface after efficient stimulation and play a role in antigen presentation to memory T cells [44,45]. In addition, a subset of MHC-II-expressing neutrophils has been recently described in bovine bone marrow and blood [46]. A subsequent report revealed the expansion of this subset of neutrophils in blood and milk of cows with bacterial mastitis [47]. In the present study, stimulation with Pam3CSK4 resulted in the upregulation of MHCII on camel neutrophils and the expansion of a MHCII^high^ fraction within camel neutrophils. The role of this subset of neutrophils in the camel immune response and the mechanism behind this selective effect of the TLR2/1 agonist should be investigated in future studies. Such studies could be designed to evaluate the capacity of Pam3CSK4-primed camel neutrophils to stimulate helper T cells in vitro.

## 5. Conclusions

Given the lack of studies on the responsiveness of camel neutrophils to bacterial and viral PAMPs, we investigated the phenotypic and functional responses of camel neutrophils after activation with synthetic ligands for TLR4, TLR 2/1, and TLR7/8. Our data indicate selective immunomodulating effects of TLR ligands on selected properties of camel neutrophils. Due to their immunomodulatory effects on the innate immune system, different TLR ligands are currently being tested as new-generation adjuvants to improve vaccine efficacy. Therefore, the findings of the current work may be supportive in vaccination studies in the dromedary camel. For the identification of immune mechanisms involved in the observed ligand-specific modulating effect on camel neutrophils, further studies are required. In addition, the impact of several animal-related factors, such as the animal’s age, gender, and breed on the observed effects should be investigated in further research.

## Data Availability

The datasets analyzed during the current study are available from the corresponding author upon reasonable request.
